# Iliac crest vertical block grafts −placing outside or inside the bone contour: A cohort study

**DOI:** 10.1111/cid.13370

**Published:** 2024-08-08

**Authors:** Christian Mertens, Christopher Büsch, Oliver Ristow, Jürgen Hoffmann, Hom‐Lay Wang, Korbinian Jochen Hoffmann

**Affiliations:** ^1^ Department of Oral‐ and Cranio‐Maxillofacial Surgery Heidelberg University Hospital Heidelberg Germany; ^2^ Institute of Medical Biometry University of Heidelberg Heidelberg Germany; ^3^ Department of Periodontics and Oral Medicine University of Michigan, School of Dentistry Ann Arbor Michigan USA

**Keywords:** alveolar ridge augmentation, bone regeneration, bone transplantation, dental implants, iliac crest bone, preprosthetic, treatment outcome

## Abstract

**Objective and Aim:**

Challenging defect configurations and dimensions arise from severe, localized vertical alveolar ridge defects caused by trauma or prior surgery. This study aims to analyze three‐dimensional bone gain, assess marginal bone stability in such defect configurations, and evaluate the impact of grafting outside the bone contour on the overall outcome, with a focus on iliac crest block grafts as a valid treatment option.

**Materials and Methods:**

The prospective cohort study evaluated patients who required vertical block grafting due to localized bone defects in the maxilla or mandible and who had received iliac grafts. Three‐dimensional bone gain was analyzed using cone beam computed tomography (CBCT) after 3 months of bone healing for each treated site and implant position. A comparison between bone grafts inside and outside the bone contour was conducted. Marginal bone stability was measured using intraoral radiographs during routine annual follow‐up visits.

**Results:**

Seventy patients with 89 treated sites were evaluated. After 3 months of graft healing, the mean vertical bone gain was 11.03 ± 3.54 mm, the mean horizontal bone gain was 7.18 ± 2.00 mm, and the mean graft length was 28.19 ± 11.01 mm. A total of 217 implants were placed in the augmented regions. On implant level, a mean vertical bone gain of 10.44 ± 3.44 mm and a mean horizontal bone gain of 6.54 ± 1.86 mm were measured. Over a 43‐month observation period, mesial and distal marginal bone loss averaged 0.44 ± 0.92 mm and 0.49 ± 1.05 mm, respectively. Eight implants were diagnosed with periimplantitis, resulting in the loss of four implants, while no early implant losses were reported.

**Conclusion:**

Within the limitations of this study, vertical bone grafts with iliac crest block grafts were found to be a dependable treatment option for dental implant placement, and placing block grafts outside the bone contour did not lead to inferior outcomes.

**Abbreviations**
BMI: Body mass index
CAD/CAM: Computer‐aided design/computer‐aided manufacturing
CBCT: Cone beam computed tomography
Chi2: Pearson's *χ* ^2^ (Chi‐squared) test
CI: Confidence Interval
F: F‐test (ANOVA)
GBR: Guided bone regeneration
KW: Kruskal‐Wallis's one‐way ANOVA
MWU: Mann–Whitney‐U‐test
PTFE: Polytetrafluoroethylene
SD: Standard Deviation
Tt2: Two sample *t* test

**Summary Box**

**What is known**

- The use of onlay block grafts from the iliac crest for bone augmentation is a well‐established procedure for treating severe alveolar ridge defects. Despite its recognized efficacy, numerous studies exhibit a gap in providing a thorough characterization of the treated defects, specifically regarding their configuration, dimensions, and location with regards to inside or outside of the bony contour.

**What this study adds**

- This study demonstrates the efficacy of iliac crest block grafts in effectively treating severe localized vertical bone defects, surpassing reported values of alternative methods and proving highly effective when grafts need placement outside the bone contour for bone arch regeneration.

## 1 INTRODUCTION

Bone defects of the alveolar ridge can vary in size and morphology and can have different etiologies. Smaller defects commonly occur due to regular tooth extraction and physiological alveolar ridge remodeling. On the other hand, larger defects may result from trauma, cystic or bone pathology removal, or even periimplantitis. These defects often exhibit a pronounced vertical component, which poses challenges in graft stabilization and soft‐tissue coverage. Moreover, an unfavorable biological aspect may arise due to the limited amount of adjacent bone. The grafting procedure can be further complicated by soft‐tissue scarring from previous surgeries.

While guided bone regeneration (GBR) is generally considered a scientifically validated approach for alveolar ridge reconstruction, patients with severe bone defects may benefit from alternative regenerative approaches. Additionally, placing bone grafts outside the natural bone contour may subject them to unfavorable pressure, potentially resulting in increased resorption of the graft material.^1^, ^2^ Nevertheless, in cases of severe defects, it may be essential to position the graft outside the bone contour to achieve complete reconstruction of the alveolar ridge. This consideration becomes especially significant in younger patients who are averse to removable restorations, as obtaining the desired fixed restoration may necessitate more extensive vertical grafting procedures.

The amount of vertical bone that can be gained with different grafting techniques may vary significantly. GBR is a successful procedure for smaller horizontal bone defects, but it has limitations in terms of vertical bone gain. A recent systematic review reported a vertical bone gain of 3.7 ± 1.4 mm.^3^ To increase the amount of bone gain, additional use of stabilization devices, such as titanium‐reinforced polytetrafluroethylene (PTFE) membranes, Computer‐Aided Design/Computer‐Aided Manufacturing (CAD/CAM) meshes, or tenting screws, is required. However, the use of these techniques increases the risk of biological complications, such as soft tissue dehiscence.^4^ For larger defects, the use of autogenous bone blocks results in more vertical bone gain. The same systematic review indicates a vertical bone gain of 5.8 ± 2.8 mm.^3^ Using the split block technique described by Khoury, even greater vertical bone gain is possible.^5^, ^6^ However, in cases where the amount of vertical bone loss is greater or the number of missing teeth is higher, intraoral bone may not always be sufficient for alveolar ridge reconstruction. In such cases, the use of iliac crest bone is still a reliable treatment option to provide nearly complete defect filling.

The efficacy of iliac crest block grafts for alveolar ridge reconstruction has been documented by numerous studies.^7^, ^8^ A recent study reported retrospective, single‐center data over a 25‐year observation period with a low complication rate and a high implant survival rate of 95% across various defect types.^8^ While studies on onlay block grafting techniques predominantly focus on complication rates and implant survival rates as primary outcome measures, there are only a few studies that comprehensively analyze bone volume gain using three‐dimensional radiographic data.

The aim of this study was to assess the bone volume gain of severe but localized bone defects in the maxilla or mandible using vertical onlay grafts from the iliac crest. The study also aimed to determine whether grafting outside the bone contour would lead to inferior outcomes with respect to bone volume gain, marginal bone loss, and complication rate.

## 2 MATERIALS AND METHODS

Ethical approval was obtained from the Ethics Committee of the Medical Faculty for this prospective, observational study (S‐075/2013). The study data were reported following STROBE (Strengthening the reporting of observation studies in epidemiology) guidelines.

The study, conducted at the Department of Oral and Craniomaxillofacial Surgery at Heidelberg University Hospital, enrolled participants from 2013 to 2021 who met predefined inclusion and exclusion criteria.

### 2.1 Inclusion criteria

- Severe localized vertical alveolar ridge defects in the maxilla or mandible (defect type 4/4 according to the Terheyden classification)^9^
- Reconstruction with vertical onlay block grafts harvested from the anterior iliac crest
- Implant placement in a two‐stage approach

### 2.2 Exclusion criteria

- Bone grafting procedures other than vertical block grafting
- Simultaneous implant placement
- Donor site other than iliac crest
- Edentulous patients
- Incomplete radiographic data (preoperative cone beam computed tomography (CBCT) and CBCT after 3 months of graft healing were the minimum required)
- Patients yet to complete their prosthetic rehabilitation
- Patients with local or systemic factors that influence bone healing (e.g., diabetes mellitus, radiation, and chemotherapy)

### 2.3 Primary outcome

The primary outcome was 3D radiographically measured vertical bone gain in millimeters after bone graft healing, as measured by CBCT.

### 2.4 Secondary outcome

- Horizontal bone gain measured on CBCT images after bone healing and before implant placement
- Marginal bone stability in mm (mesial and distal)
- Complications during the observation period
- Implant survival

### 2.5 Presurgical planning

Preoperatively, all patients with severe localized alveolar ridge defects underwent CBCT analysis to assess defect morphology and missing bone dimensions. The analysis also evaluated the mandibular–maxillary relationship and determined whether the planned bone graft would need to be placed outside the alveolar ridge bone contour. The interarch relationship was considered highly relevant. Additionally, radiographic analysis of the bone levels on the adjacent teeth was performed, as reduced bone levels limit the possibility of complete regeneration of vertical bone defects. Preoperative planning also included considerations of the final restoration, as the type of restoration can impact the decision to perform grafting procedures. If a removable restoration is a viable option for a patient with a bone defect, bone augmentations can often be avoided. However, younger patients, in particular, strongly prefer fixed restorations. If patients also wish to avoid pink porcelain or acrylics for esthetic reasons, bone augmentation procedures are unavoidable to regenerate the alveolar ridge. In addition, the patient's body mass index (BMI) was recorded.

In this study, the decision to use bone from the iliac crest was made on a case‐by‐case basis, depending on specific parameters:

- Bone defect dimensions that required more bone volume than was available from intraoral donor sites (such as retromolar area or symphysis).
- Bone defects that lacked sufficient stability for mechanical retention of particulate bone grafts.
- Bone defects requiring vertical bone grafting
- Bone defects requiring graft placement outside the bone contour or requiring compensation for interarch discrepancy
- Bone defects with an unfavorable biological defect configuration (such as a reduced amount of adjacent bone or missing walls) common in defects originating from cleft lip and palate patients or in patients who have undergone multiple previous surgeries resulting in severe soft tissue scarring.
- Insufficient residual bone that prevents other surgical procedures such as distraction devices or interpositional grafts.

### 2.6 Surgical procedure

All patients underwent treatment under general anesthesia combined with local anesthesia in the treated areas. They also received perioperative intravenous ampicillin, sulbactam antibiotics (3 g Unacid, Pfizer Pharma GmbH, Berlin, Germany), or clindamycin 600 in case of penicillin allergy.

Cortical bone blocks were harvested from the anterior iliac crest due to its higher cortical bone content^10^ and enhanced accessibility compared to the posterior iliac crest.

**FIGURE 1:**
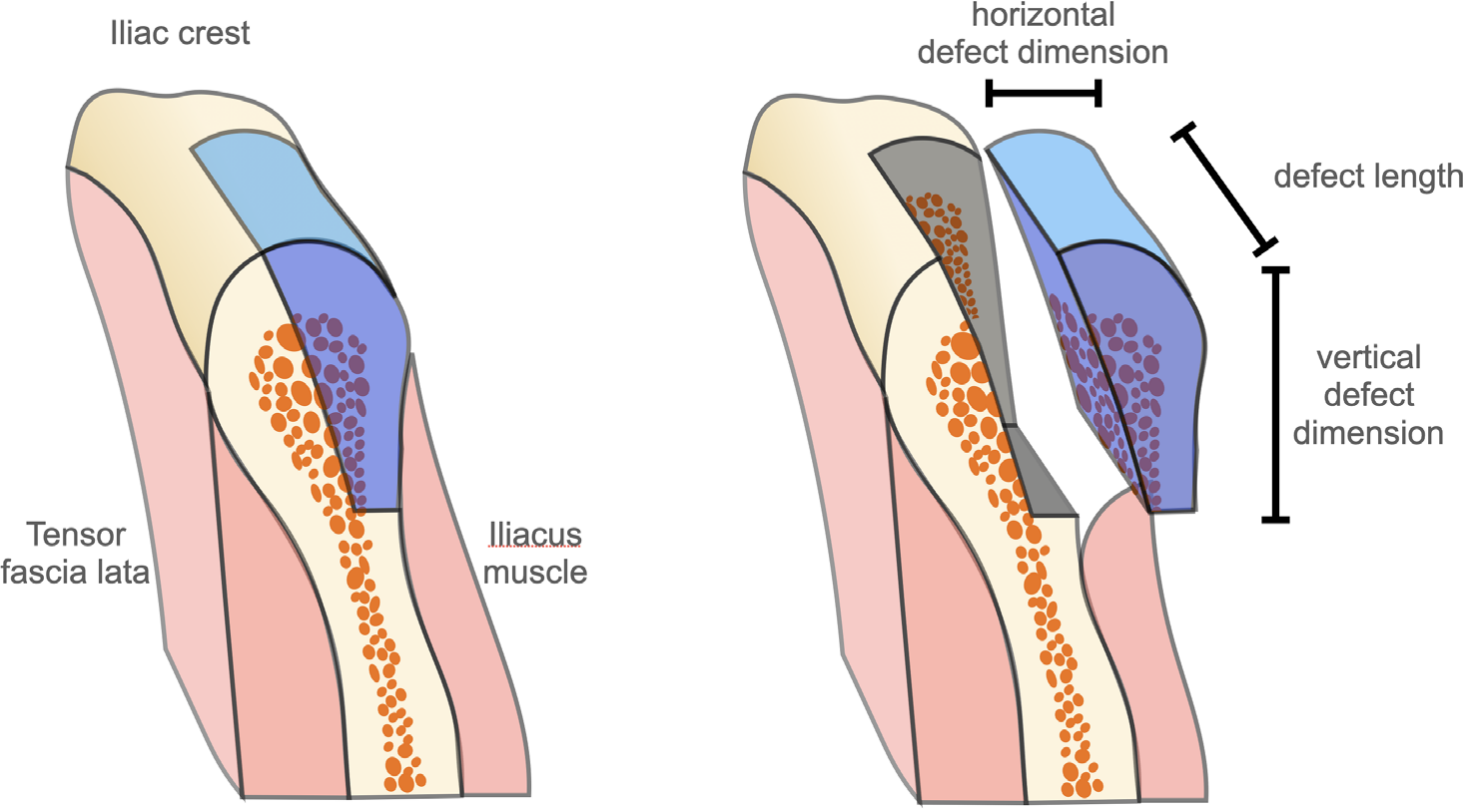
Iliac crest block harvesting from the anterior iliac crest. The annotations show the future orientation of the graft in the oral cavity.

Following an anterior–posterior incision and meticulous soft‐tissue preparation, the bony ilium was exposed according to established protocols.^11^, ^12^ Cortico‐cancellous bone blocks were then harvested from the crestal and medial regions of the ilium. The size and shape of the graft were matched to the defect size and the contour of the alveolar ridge, with a focus on maximizing cortical bone yield. The position of the superior horizontal osteotomy was based on the required graft width, while the inferior osteotomy was defined by the vertical dimension of the ridge defect (Figure 1). The anterior vertical osteotomy was placed to maintain a minimum distance of 2 cm from the anterosuperior iliac spine, and the posterior vertical osteotomy was adjusted to the length of the defect. In instances where alveolar ridge contour reconstruction was necessary, additional considerations were employed. This involved further segmentation and mitering of the harvested cortico‐cancellous bone block to effectively reconstruct the curved alveolar ridge.

**FIGURE 2:**
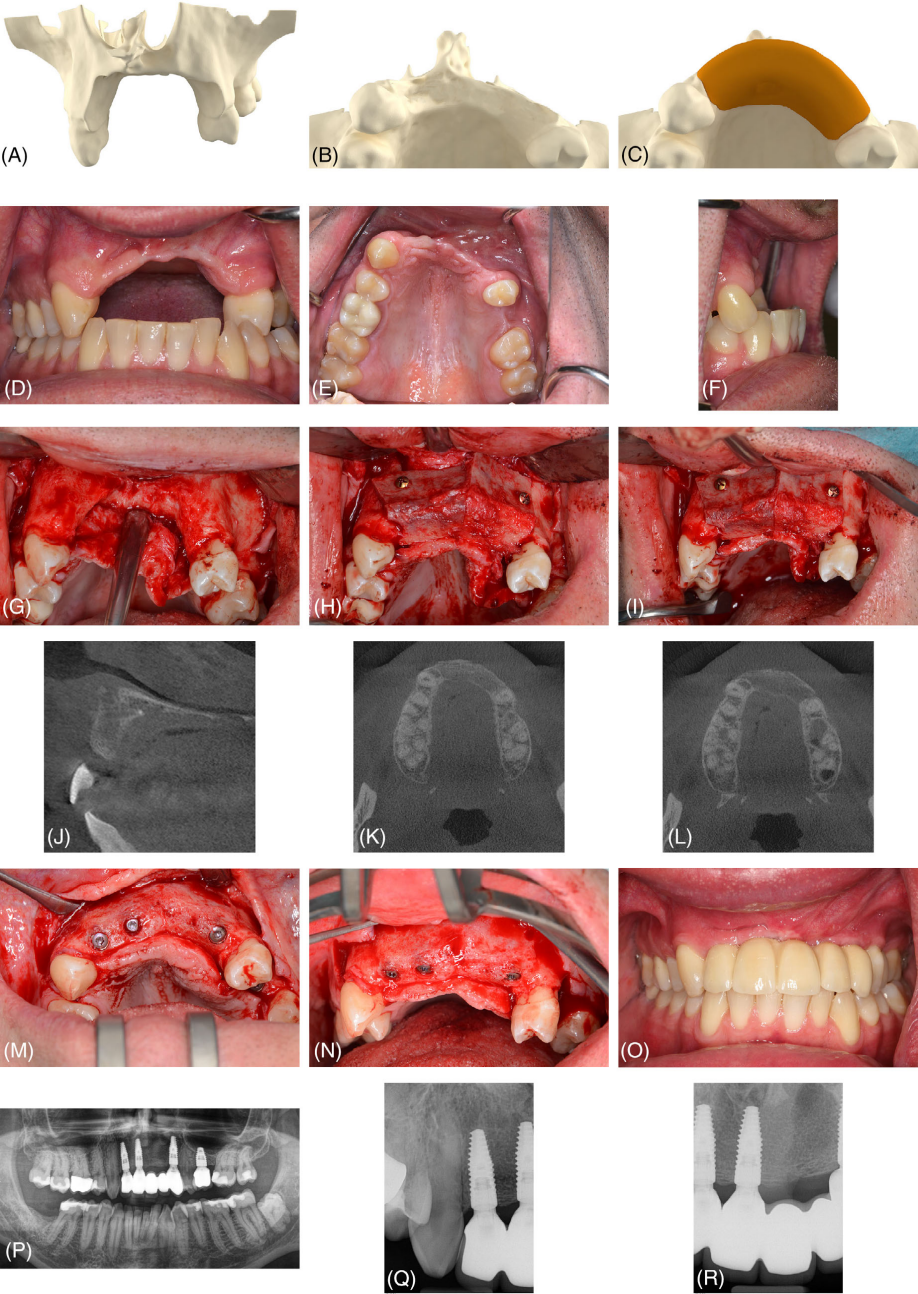
(A, B) Three‐dimensional reconstruction of the radiographic situation prior to bone augmentation; (C) determined alveolar ridge dimension; (D–F) clinical situation showing horizontal and vertical bone defect; loss of alveolar ridge contour and projection (G) intraoperative view of the defect; (H, I) situation after iliac crest block fixation with osteosynthesis screws. The cortical portions of the graft are used to reconstruct the original bone contour and the projection of the maxilla. (J–L) computed tomography images after 3 months of graft healing showing reconstructed interarch relationship (M, N) Intraoperative view at implant placement after 3 months of graft healing. The contour was fully reconstructed, and resorption was minimal. Three implants were left for submerged healing. (O) Clinical situation at follow‐up appointment, with stable soft tissue situation. Superstructure without the need to replace missing tissue with pink porcelain. (P–R) Radiographic follow‐up with stable marginal bone levels.

At the intraoral recipient site, midcrestal incisions were made in the keratinized mucosa with posterior vertical release incisions. A full‐thickness flap was reflected, and deep periosteal releasing incisions were made. The cortical portions of the iliac crest were used to reconstruct the contour of the alveolar ridge (Figure 2), while the cancellous portion was adjusted to ensure sufficient contact with the residual bone. The vertical position of the graft was determined by the bone level of the adjacent teeth. At least two titanium osteosynthesis screws were used to rigidly fix each block graft. The edges of the graft were trimmed with a round bur to prevent soft‐tissue perforation. The transition zones were filled and condensed using cancellous bone without the use of any biomaterial or membrane. Following periosteal releasing incisions and mobilization of the surrounding tissues, a tension‐free, multilayer wound closure was performed. All grafting procedures and implant insertions were carried out by a single experienced surgeon (CM).

### 2.7 Postsurgical procedure

Patients were advised to refrain from wearing their temporary prosthesis during the first 3 weeks after surgery to avoid contact with the soft tissues of the recipient site. Additionally, patients were instructed to rinse with 0.2% chlorhexidine gluconate for 7 days. Sutures were removed from the recipient site after 10–14 days.

### 2.8 Implant placement

In cases of uneventful healing, implant placement was planned 3 months after graft healing with a CBCT scan. Implant surgery was performed under local anesthesia shortly thereafter. Osteosynthesis screws were removed during the same procedure before preparing the implant site. All implants placed were bone‐level implants placed in an epicrestal position. The length of the implants was determined by the available vertical bone volume and associated anatomical structures, such as the maxillary sinus and alveolar nerve. The diameter of the implant was determined by the available horizontal bone dimension, the distance to adjacent teeth, and the position of implant placement, such as the dimension of the future crown.

### 2.9 Radiographic evaluation

**FIGURE 3:**
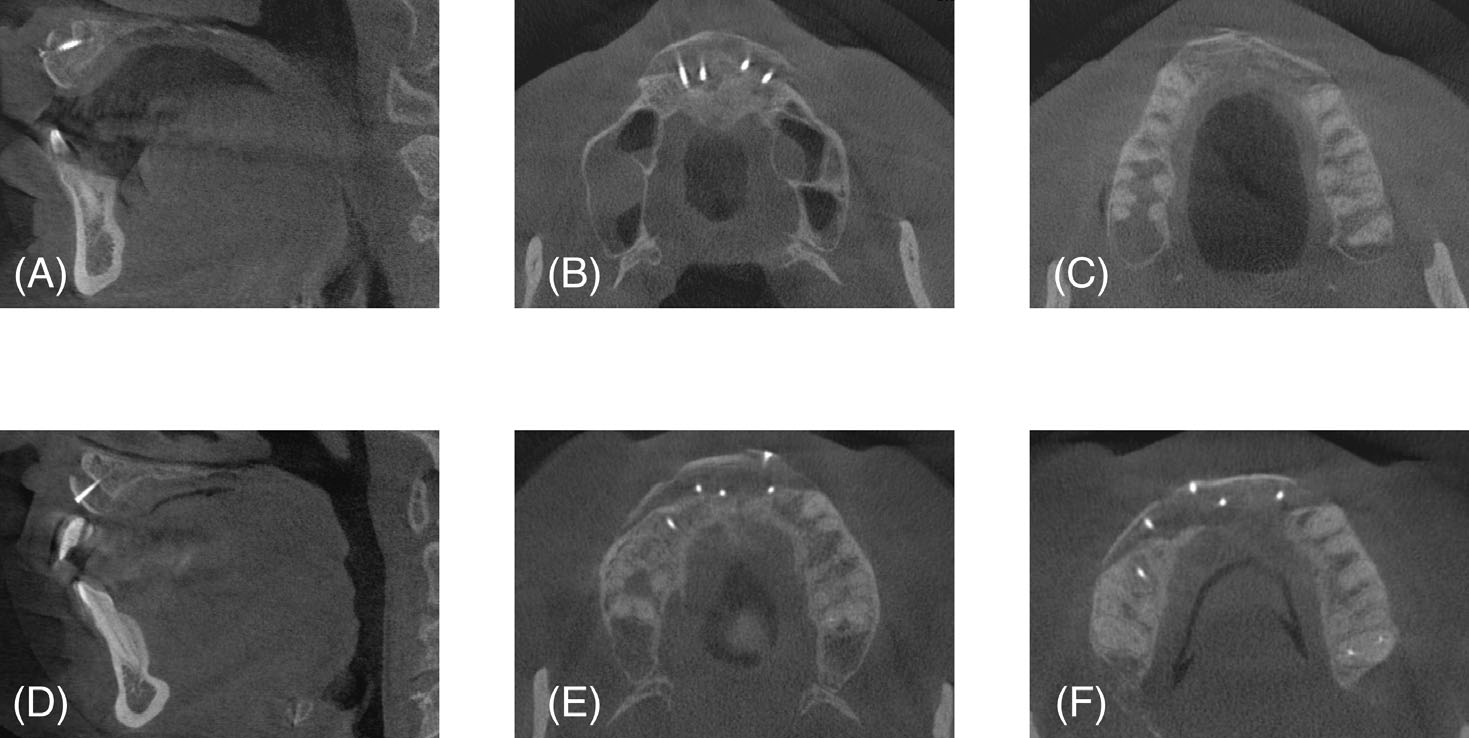
Computed tomography images of two patient cases 3 months after graft healing (A–C) patient case with reconstructed alveolar ridge in the anterior maxilla, (D–F) sagittal and axial images from a second patient case following alveolar ridge reconstruction.

A CBCT scan was taken during preoperative planning to assess defect morphology and adjacent anatomic structures (T0). After 3 months of graft healing, a second CBCT scan (T1) was taken for implant planning to analyze bone gain in the horizontal and vertical dimensions (Figure 3). The analysis of three‐dimensional bone gain was based on the two DICOM data sets, which were aligned using a three‐point matching procedure with the teeth as a reference to ensure correct alignment.

The dimensions of each block graft were measured at the “graft level” with the following parameters analyzed: height, width, and length in millimeters. The bone gain of each prospective implant position was also measured. The parameters vertical and horizontal bone gain were evaluated.

Marginal bone levels were assessed through intraoral radiographs during annual follow‐up visits. The implants were utilized as reference objects to correct dimensional deviations. The distance from the implant shoulder to the first bone contact to the bone on the epicrestally placed implants was measured.

All measurements were performed by a single calibrated examiner who was not actively involved in the treatment of the patients.

### 2.10 Complications

Complications were recorded throughout the observation period. These included both donor and recipient site complications. Recipient site complications comprised postoperative wound infection, soft‐tissue dehiscence, graft exposure, lack of graft stability, neurological disorders, and bone graft loss. Severe bone graft resorption was also highly relevant.

Donor site complications included postoperative infection, dehiscence, and iliac crest fracture.

### 2.11 Statistical methods

Continuous variables were expressed as the mean ± SD (standard deviation), and categorical variables as absolute and relative frequencies. Appropriate testing of the distribution of continuous variables and corresponding tests (student's *t*‐test or Mann‐Whitney‐U‐test) were performed. For categorical variables, Pearson's *χ* ^2^ test was used. In addition, the number of missing values was added (if present).

Mean vertical bone gain, mean horizontal bone gain, implant diameter, and implant length are variables at the implant level and were hence compared using a univariate linear mixed model with sagittal (inside /outside bone contour) as fixed effect and nested random effect for patient and graft.

The primary objective, to ascertain whether different clinical parameters influence vertical bone gain, was investigated using a linear mixed effect model. As a dependent variable, the outcome of vertical bone gain was used. As fixed effects (independent variables), the implant diameter (in mm), implant length (in mm), age at augmentation (in years), number of missing teeth, indication (trauma/atrophy/cleft palate/bone pathology), BMI at augmentation, and jaw (maxilla/mandible) were added to the model. Furthermore, nested random effects for patient and graft were added to the model to account for patient and graft heterogeneity.

The secondary outcomes, horizontal bone gain and marginal bone stability (mesial and distal), were analyzed using similar models as for the primary outcome.

To investigate whether the extent and dimensions of the bone graft influenced marginal bone loss, graft length, width, and height measured at graft level were additionally added as fixed effects for the models of mesial and distal marginal bone stability.

Due to the exploratory characteristics of this trial, the *p*‐values were not adjusted for multiplicity, and a *p*‐value of less than 0.05 can be regarded as significant. Statistical analyses were conducted using the statistic software R (version 4.2.1, R Core Team, Auckland, New Zealand) using the packages “lme4” and “lmerTest” for linear mixed effect models and “ggplot2” for data illustrations.

## 3 RESULTS

### 3.1 Patient level

The study included 70 patients who underwent 89 vertical onlay bone grafts sourced from the iliac crest. All defects were classified as class 4/4 according to the Terheyden classification.^9^

The mean age at bone grafting was 44.55 ± 15.51 years (Table 1). The mean Body Mass Index (BMI) was 25.72 ± 4.82. The mean defect size was 4.68 ± 2.52 missing teeth. Among the patients, 29 (42.65%) had one to three missing teeth, 25 (36.76%) had four to six missing teeth, 14 (20.59%) had more than six missing teeth, and two (2.86%) had missing values.

**TABLE 1:** Patient demographics grouped by “missing teeth numbers—3 Levels.”

Variable	1–3	4–6	>6	Total	*p*
	(*N* = 29)	(*N* = 25)	(*N* = 14)	(*N* = 68)	
Indication					
Trauma	3 (10.34%)	9 (36.00%)	4 (28.57%)	16 (23.53%)	
Atrophy	16 (55.17%)	10 (40.00%)	7 (50.00%)	33 (48.53%)	
Cleft palate	4 (13.79%)	3 (12.00%)	3 (21.43%)	10 (14.71%)	
Pathology	6 (20.69%)	3 (12.00%)	0 (0.00%)	9 (13.24%)	
Age at augmentation					
N	29	25	14	68	0.393^KW^
Mean ± SD	43.66 ± 15.72	43.12 ± 14.88	48.93 ± 16.51	44.55 ± 15.51	
Median (Q1, Q3)	46.48 (31.74, 54.74)	45.99 (31.86, 54.73)	54.28 (39.76, 61.50)	47.02 (31.80, 56.28)	
Min–max	15.8–69.5	18.1–71	19.7–65.7	15.8–71	
Number of missing teeth					
*N*	29	25	14	68	
Mean ± SD	2.621 ± 0.49	4.800 ± 0.82	8.714 ± 1.978	4.676 ± 2.518	
Median (Q1, Q3)	3.000 (2.000, 3.000)	5.000 (4.000, 5.000)	8.500 (7.000, 9.000)	4.000 (3.000, 6.000)	
Min–max	2–3	4–6	7–14	2–14	
BMI					
*N*	28	24	14	66	0.721^F^
Nmiss	1 (3.45%)	1 (4.00%)	0 (0.00%)	2 (2.94%)	
Mean ± SD	25.21 ± 5.413	25.89 ± 5.056	26.46 ± 2.989	25.72 ± 4.821	
Median (Q1, Q3)	24.80 (21.10, 28.85)	24.50 (22.85, 28.85)	27.25 (24.20, 28.10)	25.00 (23.00, 28.70)	
Min–max	16–37.6	18.7–37	20.4–31.2	16–37.6	
Regrafting					
No	20 (68.97%)	19 (76.00%)	11 (78.57%)	50 (73.53%)	0.751^chi2^
Yes	9 (31.03%)	6 (24.00%)	3 (21.43%)	18 (26.47%)	
Prosthetic rehabilitation					
Fixed	29 (100.00%)	25 (100.00%)	11 (78.57%)	65 (95.59%)	
Removable	0 (0.00%)	0 (0.00%)	3 (21.43%)	3 (4.41%)	
Pink porcelain					
No	28 (96.55%)	19 (76.00%)	8 (57.14%)	55 (80.88%)	0.006^chi2^
Yes	1 (3.45%)	6 (24.00%)	6 (42.86%)	13 (19.12%)	

*Note*: ^chi2^ Pearson's chi‐squared test, ^KW^ Kruskal‐Wallis's one‐way ANOVA, ^F^ F‐test (ANOVA).

Of the final implant‐retained restorations, the majority were fixed (*n* = 65, 95.59%), with only a few being removable (*n* = 3, 4.41%). Moreover, 10 of the fixed restorations required the use of pink porcelain (Table 1).

### 3.2 Graft level

A total of 45 grafts (51.14%) were placed in anterior zones and 43 grafts (48.86%) in posterior zones. Thirty‐seven block grafts (41.57%) were placed in the maxilla, while 52 (58.43%) were in the mandible.

At the graft level, the mean vertical bone gain was 11.03 mm ± 3.54, the mean horizontal bone gain was 7.18 mm ± 2.00, and the mean graft length was 28.19 mm ± 11.01. In the maxilla, the mean vertical bone gain was 12.14 mm ± 3.38, the mean horizontal bone gain was 7.16 mm ± 1.92, and the mean graft length was 27.14 mm ± 11.76. In the mandible, the mean vertical bone gain was 10.24 mm ± 3.46, the mean horizontal bone gain was 7.20 mm ± 2.07, and the mean graft length was 28.95 mm ± 10.49. Notably, vertical bone gain was significantly higher in the maxilla than in the mandible (*p* ^MWU^ = 0.006). Recipient site complications did not show a significant difference between the mandible and maxilla (*p* ^chi2^ = 0.878) (Table 2).

**TABLE 2:** Description of data on graft level grouped by maxilla and mandible.

Variable	Maxilla	Mandible	Total	*p*
	(*N* = 37)	(*N* = 52)	(*N* = 89)	
Graft length				
*N*	37	52	89	0.130^MWU^
Mean ± SD	27.14 ± 11.76	28.95 ± 10.49	28.19 ± 11.01	
Median (Q1, Q3)	24.01 (18.90, 34.82)	28.47 (23.14, 32.13)	26.66 (20.75, 32.70)	
Min–max	7.81–62.1	11.2–60.8	7.81–62.1	
Graft width				
*N*	37	52	89	0.777^MWU^
Mean ± SD	7.164 ± 1.920	7.198 ± 2.071	7.184 ± 1.999	
Median (Q1, Q3)	6.780 (5.660, 8.400)	7.315 (5.700, 8.085)	6.950 (5.660, 8.110)	
Min–max	4.06–11.3	2.49–13.1	2.49–13.1	
Graft height				
*N*	37	52	89	0.006^MWU^
Mean ± SD	12.14 ± 3.383	10.24 ± 3.462	11.03 ± 3.539	
Median (Q1, Q3)	12.81 (10.47, 14.58)	10.27 (7.835, 12.26)	10.75 (8.750, 13.79)	
Min–max	3.71–17.4	3.3–18.9	3.3–18.9	
Contour				
Inside	10 (27.78%)	35 (67.31%)	45 (51.14%)	<0.001^chi2^
Outside	26 (72.22%)	17 (32.69%)	43 (48.86%)	
(Missing)	1 (2.70%)	0 (0.00%)	1 (1.12%)	
Region				
Anterior	27 (72.97%)	19 (36.54%)	46 (51.69%)	<0.001^chi2^
Posterior	10 (27.03%)	33 (63.46%)	43 (48.31%)	
Complications recipient site				
No complications	32 (86.49%)	43 (82.69%)	75 (84.27%)	0.878^chi2^
Infection	2 (5.41%)	4 (7.69%)	6 (6.74%)	
Dehiscence	3 (8.11%)	5 (9.62%)	8 (8.99%)	
Regrafting				
No regrafting	36 (97.30%)	48 (92.31%)	84 (94.38%)	0.458^chi2^
GBR	1 (2.70%)	2 (3.85%)	3 (3.37%)	
Retromolar bone	0 (0.00%)	2 (3.85%)	2 (2.25%)	

*Note*: ^MWU^Mann‐Whitney's U test, ^chi2^Pearson's chi‐squared test.

Bone grafts placed outside the bone contour to compensate for interarch discrepancy were primarily located in the anterior region (*n* = 42, 97.67%). Of these, 60.5% were located in the maxilla and 39.5% in the mandible. The vertical bone gain for this indication was 13.29 mm ± 2.60 and the horizontal bone gain was 7.25 mm ± 1.71. In contrast, bone grafts that did not need to compensate for interarch discrepancy had a vertical bone gain of 8.98 mm ± 2.96 and a horizontal bone gain of 7.16 mm ± 2.26. Vertical bone gain was significantly higher when bone grafts were placed outside the bone contour (*p* < 0.001) (Table 3).

**TABLE 3:** Description of data on graft level grouped by inside and outside the bone contour.

Variable	Inside contour (*N* = 45)	Outside contour (*N* = 43)	Total (*N* = 88)	*p*
Graft length				
*N*	45	43	88	0.822^MWU^
Mean ± SD	27.19 ± 8.351	29.47 ± 13.26	28.30 ± 11.02	
Median (Q1, Q3)	27.61 (23.38, 29.52)	26.40 (19.02, 36.44)	26.66 (20.76, 33.22)	
Min–max	11.2–57.4	7.81–62.1	7.81–62.1	
Graft width				
*N*	45	43	88	0.602^MWU^
Mean ± SD	7.160 ± 2.264	7.250 ± 1.710	7.204 ± 2.001	
Median (Q1, Q3)	7.220 (5.470, 7.960)	6.930 (6.160, 8.750)	7.015 (5.710, 8.255)	
Min–max	2.49–13.1	3.01–11.3	2.49–13.1	
Graft height				
*N*	45	43	88	<0.001^MWU^
Mean ± Sd	8.978 ± 2.962	13.29 ± 2.598	11.08 ± 3.520	
Median (Q1, Q3)	9.110 (7.220, 10.63)	13.33 (11.04, 15.30)	10.76 (8.870, 13.90)	
Min–max	3.3–16.6	8–18.9	3.3–18.9	
Region				
Anterior	3 (6.67%)	42 (97.67%)	45 (51.14%)	<0.001^chi2^
Posterior	42 (93.33%)	1 (2.33%)	43 (48.86%)	
Jaw				
Maxilla	10 (22.22%)	26 (60.47%)	36 (40.91%)	<0.001^chi2^
Mandible	35 (77.78%)	17 (39.53%)	52 (59.09%)	
Complication recipient site				
No complications	37 (82.22%)	37 (86.05%)	74 (84.09%)	0.733^chi2^
Infection	4 (8.89%)	2 (4.65%)	6 (6.82%)	
Dehiscence	4 (8.89%)	4 (9.30%)	8 (9.09%)	
Regrafting				
No regrafting	43 (95.56%)	40 (93.02%)	83 (94.32%)	0.820^chi2^
GBR	1 (2.22%)	2 (4.65%)	3 (3.41%)	
Retromolar bone	1 (2.22%)	1 (2.33%)	2 (2.27%)	

*Note*: ^MWU^ Mann‐Whitney's U test, ^chi2^ Pearson's chi‐squared test.

The placement of bone grafts outside the bone contour did not result in a higher rate of recipient site complication (postoperative infection: *n* = 2 [4.65%] vs. *n* = 4 [8.89%]; dehiscence rate: *n* = 4 [9.30%] vs. *n* = 4 [8.89%]).

Importantly, the defect size, indicating by the number of missing teeth, did not influence the amount of vertical bone gain.

Regrafting at implant placement was required in five cases (GBR = 3, intraoral bone block = 2).

### 3.3 Implant level

**FIGURE 4:**
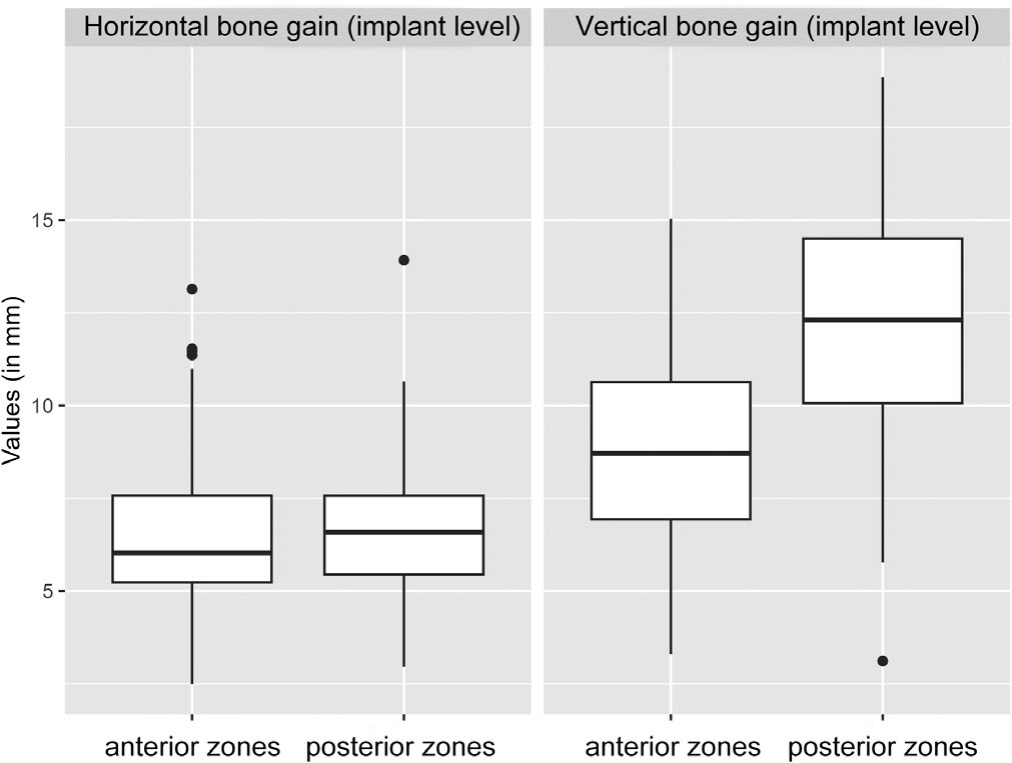
Horizontal and vertical bone gain in mm grouped by anterior/posterior zones.

The mean vertical bone gain at the implant level was 10.44 mm ± 3.44, while the mean horizontal bone gain was 6.54 mm ± 1.86 (Table 4). Figure 4 displays the vertical and horizontal bone gain for the anterior and posterior zones.

**TABLE 4:** Description of data on implant level grouped by inside and outside the bone contour.

Variable	Inside contour	Outside contour	Total	*p*
	(*N* = 105)	(*N* = 112)	(*N* = 217)	
Marginal bone loss mesial				
*N*	102	110	212	0.006^MWU^
Nmiss	3 (2.86%)	2 (1.79%)	5 (2.30%)	
Mean ± SD	0.62 ± 1.077	0.28 ± 0.70	0.44 ± 0.92	
Median (Q1, Q3)	0 (0, 0.90)	0 (0, 0.31)	0 (0, 0.59)	
Min–max	0–6.74	0–5.46	0–6.74	
Marginal bone loss distal				
*N*	102	110	212	<0.001^MWU^
Nmiss	3 (2.86%)	2 (1.79%)	5 (2.30%)	
Mean ± SD	0.77 ± 1.306	0.23 ± 0.64	0.49 ± 1.050	
Median (Q1, Q3)	0 (0, 1.070)	0 (0, 0)	0 (0, 0.67)	
Min–max	0–6.85	0–5.7	0–6.85	
Complications				
No complications	99 (94.29%)	110 (98.21%)	209 (96.31%)	
Periimplantitis	4 (3.81%)	0 (0.00%)	4 (1.84%)	
Implant loss	2 (1.90%)	2 (1.79%)	4 (1.84%)	
Implant diameter				
*N*	105	112	217	0.01^MWU^
Mean ± SD	4.019 ± 0.37	3.904 ± 0.37	3.959 ± 0.38	
Median (Q1, Q3)	4.200 (3.600, 4.200)	3.600 (3.600, 4.200)	4.100 (3.600, 4.200)	
Min–max	3.5–4.8	3.3–4.8	3.3–4.8	
Implant length				
*N*	105	112	217	<0.001^MWU^
Mean ± SD	9.933 ± 1.469	11.23 ± 1.065	10.60 ± 1.430	
Median (Q1, Q3)	10.00 (9.000, 11.00)	11.00 (11.00, 11.00)	11.00 (9.000, 11.00)	
Min–max	6–13	8–13	6–13	
Implant brand				
Astra	97 (92.38%)	103 (91.96%)	200 (92.17%)	0.364^chi2^
Straumann	8 (7.62%)	7 (6.25%)	15 (6.91%)	
Camlog	0 (0.00%)	2 (1.79%)	2 (0.92%)	
Graft width				
*N*	101	108	209	0.372^tt2^
Nmiss	4 (3.81%)	4 (3.57%)	8 (3.69%)	
Mean ± SD	6.423 ± 1.920	6.654 ± 1.799	6.542 ± 1.858	
Median (Q1, Q3)	6.030 (5.230, 7.570)	6.580 (5.430, 7.585)	6.390 (5.250, 7.570)	
Min–max	2.49–13.1	2.95–13.9	2.49–13.9	
Graft height				
*N*	101	108	209	<0.001^tt2^
Nmiss	4 (3.81%)	4 (3.57%)	8 (3.69%)	
Mean ± SD	8.683 ± 2.775	12.09 ± 3.177	10.44 ± 3.436	
Median (Q1, Q3)	8.710 (6.930, 10.63)	12.31 (10.06, 14.51)	10.47 (7.990, 13.07)	
Min–max	3.3–15	3.11–18.9	3.11–18.9	

*Note*: ^MWU^ Mann‐Whitney's U test, ^chi2^ Pearson's chi‐squared test.

**FIGURE 5:**
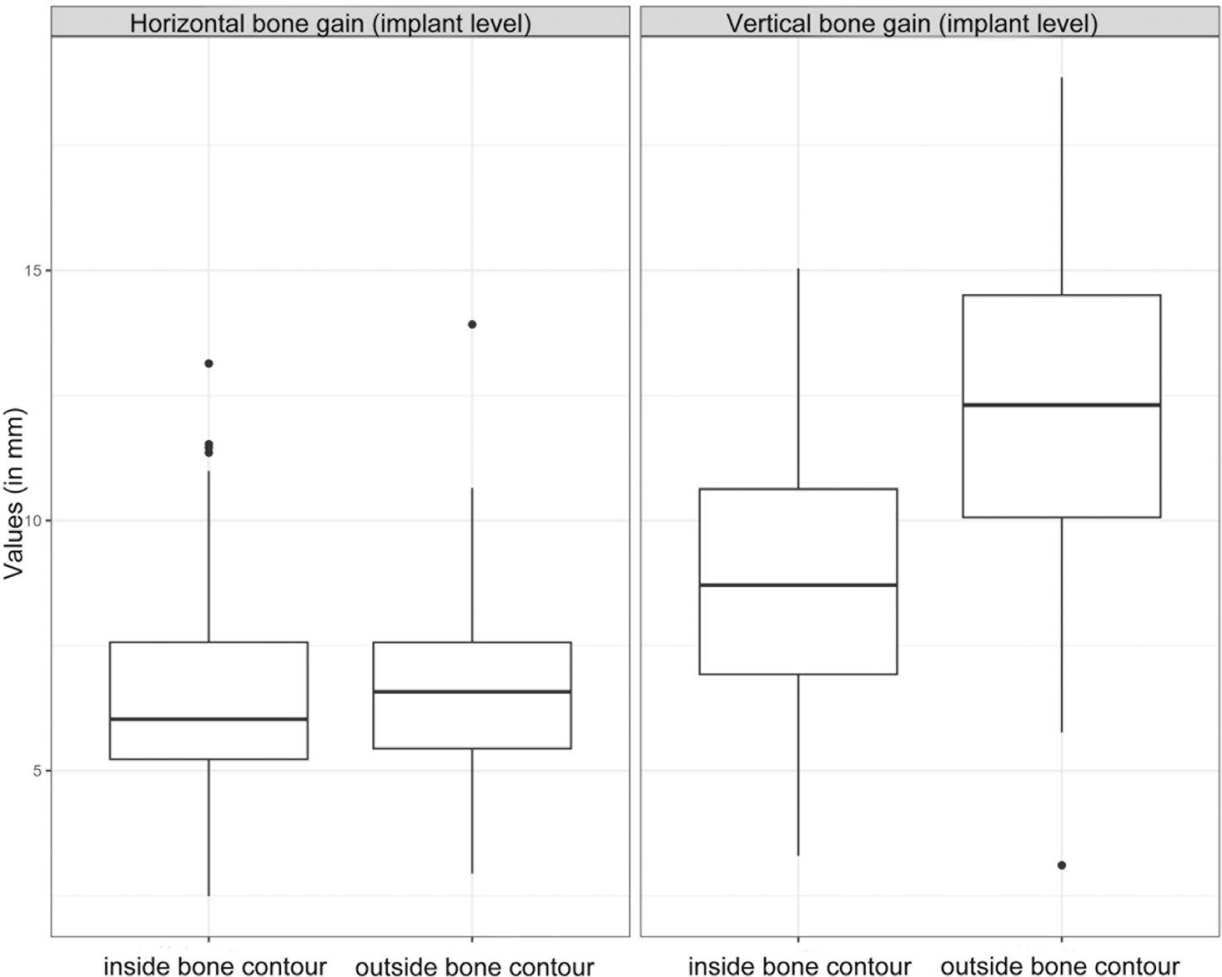
Horizontal and vertical bone gain in mm grouped by inside versus outside bone contour.

Bone grafts placed outside the bone contour resulted in a 0.2 mm higher horizontal bone gain (95% CI: [−0.52, 0.91], *p* = 0.588) and a 3.21 mm higher vertical bone gain (95% CI: [2.02, 4.40], *p* < 0.001) than bone grafts placed inside the bone contour (Figure 5).

For implant diameter, the difference between outside and inside the bone contour was 1.03 mm (95% CI: [0.44, 1.63], *p* = 0.001), whereas implant length did not show a significant difference of −0.12 (95% CI: [−0.24, 0.01], *p* = 0.071).

In the linear mixed regression model (*n* = 197), the indication “trauma” showed significantly better results in terms of the achieved graft height. Comparing grafts in the “trauma” and “atrophy” groups, the mean augmented bone height in the “atrophy” group was 1.953 mm (95% CI: [−3.481, −0.348], *p* = 0.025) smaller than for a graft in the “trauma” group. No statistically significant effect could be seen regarding the influence of graft width (95% CI: [−0.591, 1.326], *p* = 0.478).

**FIGURE 6:**
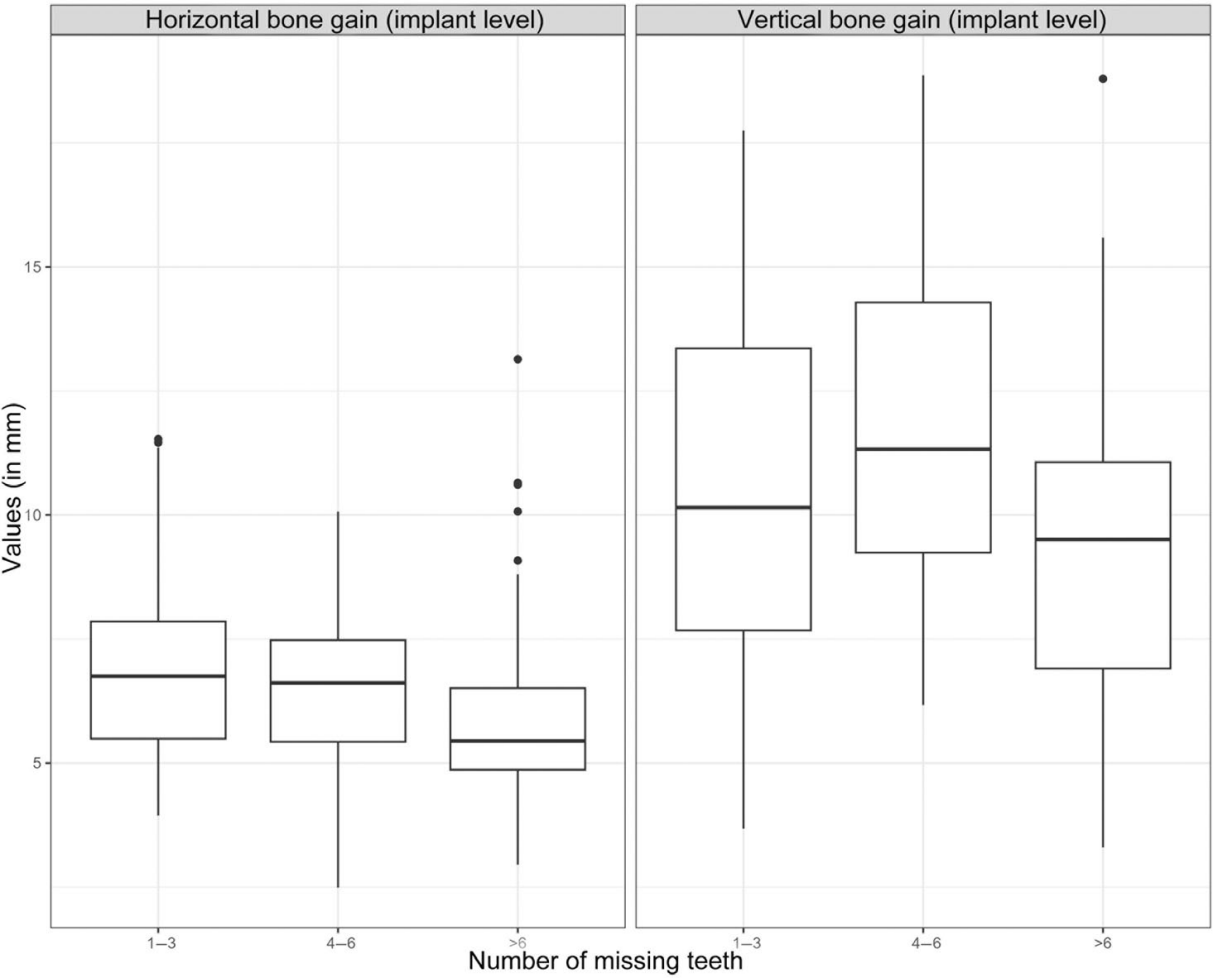
Horizontal and vertical bone gain in mm grouped by number of missing teeths.

Furthermore, there was a statistically significant difference in graft height when considering augmentation in the maxilla or mandible. Comparing two grafts in the “maxilla” and “mandible” group, the mean augmented bone height in the “mandible” group was 1.245 mm (95% CI: [−2.307, −0.091], *p* = 0.037) smaller than for a graft in the “maxilla” group. A statistically‐significant affect could be seen on the influence of graft height (95% CI: [−0.12, 1.185], *p* = 0.039). Other data showed no significant influence on graft height and width, but it should be mentioned that an increase in missing teeth showed a slight decrease in graft width {−0.102 mm per missing tooth (95% CI: [−0.233, 0.022], *p* = 0.138)} and graft height {−0.199 mm per missing tooth (95% CI: [−0.418, 0.016], *p* = 0.101)} (Figure 6 and Appendix, Tables A1 and A2).

A total of 217 implants were placed in sites with augmented bone volume. Implant placement was possible in all cases due to sufficient bone volume. One hundred‐two implants were placed in anterior sites and 117 in posterior sites. In terms of location, 91 implants were placed in the maxilla and 128 in the mandible. During the observation period, 209 implants did not show any complications (96.31%). Four implants (1.84%) were lost, and four implants (1.84%) developed periimplantitis. Of the four lost implants, two were placed outside the contour, and two implants were placed inside the contour. The four cases with periimplantitis were all located inside the contour.

**FIGURE 7:**
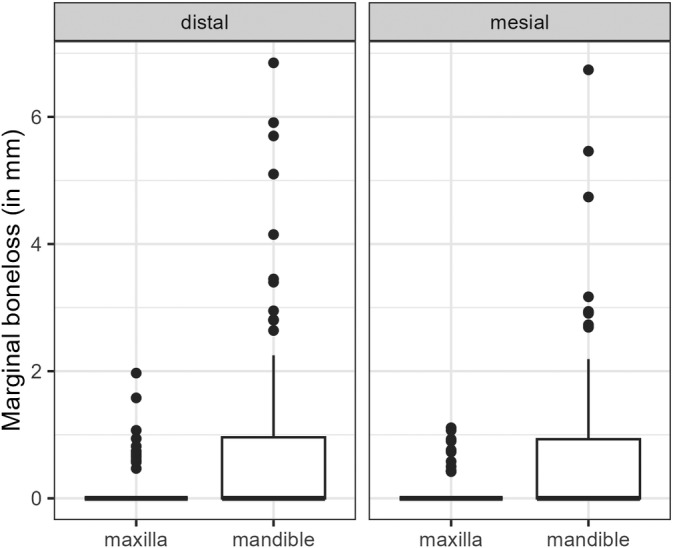
Marginal bone loss observed in the mesial and distal sites of the implants (in mm) grouped by maxilla/mandible.

The mean mesial marginal bone loss was 0.44 mm ± 0.92, and the mean distal marginal bone loss was 0.49 mm ± 1.05 after a mean follow‐up of 42.98 months ±32.62. Marginal bone loss was not significantly affected by implant diameter, implant length, patient age at augmentation, BMI, indication, or defect size. Furthermore, the linear mixed regression models showed no statistically significant influence of the amount of vertical or horizontal bone gained on marginal bone loss. The treated jaw, however, showed a statistically significant impact on marginal bone loss, with the mandible showing significantly higher marginal bone loss than the maxilla {mesial: 0.5019 mm (95% CI: [0.153, 0.850], *p* = 0.006); distal: + 0.476 (95% CI: [0.0982, 0.854], *p* = 0.015)} (Figure 7 and Appendix, Tables A3 and A4).

## 4 DISCUSSION

The present study demonstrates that vertical bone grafts with iliac crest block grafts are a reliable treatment option for dental implant placement, and placing block grafts outside the bone contour does not lead to inferior outcomes. In summary, this study revealed a vertical bone gain of 11.03 mm ± 3.54 at the graft level, with a complication rate of 15.91% (*n* = 14), including 6.82% (*n* = 6) postoperative infections and 9.09% (*n* = 8) postoperative dehiscence when utilizing iliac crest block grafts. All sites healed successfully after conservative management with local treatment and allowed for implant placement. No graft failure was observed.

In a comprehensive retrospective analysis of 190 cases involving different horizontal and vertical bone defects treated with iliac crest onlay block grafts, recipient site complications were observed at a rate of 21.58%: graft loss (*n* = 2), postoperative dehiscence (*n* = 13), minor sequestra (*n* = 9), and localized infection (*n* = 17). The primary outcome in this study, however, was implant survival, not bone gain.^8^

Localized, severe vertical bone defects present a significant challenge in implant treatment. While various treatment approaches are available, they can yield different rates of bone gain and complications. A recent systematic review compared several techniques,^4^ including distraction osteogenesis, which showed a vertical bone gain of 8.04 mm but a high complication rate of 47.3%. On the other hand, GBR resulted in a bone gain of 4.18 mm with a lower complication rate of 12.1%. When absorbable membranes were exclusively utilized, the vertical bone gain was 3.51 mm, while non‐resorbable membranes led to a gain of 4.42 mm. The use of autogenous bone blocks resulted in a bone gain of 4.12 mm, albeit with a complication rate of 23.9%.^4^

Iliac crest onlay block grafts are typically employed for the augmentation of substantial bone defects, particularly in cases of severely resorbed jaws or vertical bone defects.^7^, ^8^ The anterior iliac crest, provides a thick cortical layer, that is ideal for alveolar ridge reconstruction.^11^ A recent randomized controlled clinical trial compared the histomorphological and radiographic characteristics of calvarial and anterior iliac crest bone grafts for pre‐implant procedures, finding both graft types well‐incorporated after 4 months.^12^ Calvarial grafts exhibited less resorption over time and higher mineral density compared to iliac crest grafts, while the latter showed higher metabolic activity. The authors concluded that both types of graft were capable of providing a stable base for implant placement. The implant survival rate at 1 year was 97.8%.

Although associated with a higher incidence of donor‐site morbidity compared to intraoral donor sites, such complications are typically limited to the initial postoperative weeks.^13^ The size of the harvested graft, however, influences postoperative morbidity such as gait disturbances.

Implant placement can be performed after 3 months of healing, representing a duration half that of vertical guided bone regeneration procedures. Employing a staged implant placement approach, studies show a high rate of implant survival, and the maintenance of marginal bone stability exhibits sustained efficacy over an extended observation period.^14^

This study results indicate that the employed technique is capable of generating substantial vertical bone and facilitating the reconstruction of the bone contour, even when the graft necessitates placement outside the original bone contour. More bone gain was observed in cases where the bone block was positioned outside the contour without increasing the complication rates or marginal bone loss. Onlay block grafts from the iliac crest may be a reliable and effective treatment option for these types of defects.

These findings are in contrast to a recent umbrella review from 2023, which showed that greater marginal bone loss was observed with longer implants after augmentation compared to short implants in native bone.^15^ Esposito et al. showed similar results in a review article from 2019, where longer implants in augmented bone also showed a higher mean marginal bone loss (mean difference = 0.60 mm; 95% CI: 0.36–0.83; *p* < 0.00001; *I* ^2^ = 45%).^16^ However, neither of the studies differentiated between the type of bone augmentation and the type of defect. In addition, the risk of bias in the included studies was high.

As observed, vertical bone grafting procedures typically exhibit higher complication rates compared to horizontal bone grafting. Nevertheless, the onlay block technique stands out for its remarkable versatility, accommodating a wide range of indications. In contrast, alternative methods, such as distraction osteogenesis, are constrained to larger segments, necessitate a specific amount of residual bone, and lack the capability to simultaneously create horizontal bone gain. Postoperative complications can lead to loss of the osteotomized bone segment, worsening the initial bone defect. Additionally, the vector can be compromised during elongation, or basal bone fractures can occur.^17^

The use of particulate grafts in vertical bone augmentation will result in unfavorable graft stability, leading to extensive resorption and buccal bone remodeling. Recent publications highlighted this problem already with smaller defects.^1^, ^2^ Gao et al. compared outside‐contour and inside‐contour groups, and found a statistically significant difference with more remodeling in the outside bone contour group. However, this effect could also be a consequence of the particulate material used in the study. Xi et al. even state that new bone formation with GBR procedures at the coronal site may only be predictable if the graft is placed within the bony envelope.^2^ Thus, GBR procedures require additional stabilization in the form of titanium‐reinforced membranes to be successful when placed in non‐contained defects.^18^ Vertical defects are even more challenging, and the vertical bone gain seems to be limited to a certain dimension, as shown by Urban et al., who reported 5.5 mm ± 2.29 mm in a retrospective study.^19^ The use of CAD/CAM meshes appears to be a promising technique with minimal adaptation time needed to fit the graft to the recipient site. A recent retrospective study demonstrated a mean vertical bone gain of 4.78 ± 1.88 mm with a mesh exposure rate of 20.75%.^20^ However, when using smaller autogenous bone blocks for augmentation procedures, it was again found that blocks placed outside the bone contour resulted in more bone resorption compared to blocks placed inside the bone contour after 3 months of healing.^21^

The results of this study indicate that greater vertical bone augmentation does not correlate with higher complication rates or a greater marginal bone loss. The impact of greater vertical bone augmentation on marginal bone loss appears negligible, as evidenced by an estimate close to zero {mesial: −0.0301 mm (95% CI: [−0.1254, 0.0652], *p* = 0.5203); distal: −0.0448 (95% CI: [0.1356, 0.0460], *p* = 0.3165)}. It is noteworthy that low bone resorption may be attributed to adjacent bone and teeth, with the adjacent bone, rather than the initially grafted bone amount, determining the long‐term maximum vertical bone level. This observation may elucidate why patients with bone atrophy achieve less bone height than those with trauma, given the fact that general bone loss is usually observed in bone atrophy patients. The inclusion criteria of this study excluded edentulous patients. It is unlikely that vertical block augmentation in these patients would achieve the same amount of vertical bone gain as described with the localized ridge defects.

The surgical incision design of the patients treated was conventional mid‐crestal incisions combined with vertical releasing incisions. In a retrospective analysis examining complications of mandibular ramus block grafts, crestal incisions were found to correlate with an increased risk of early postoperative complications.^22^ Some studies suggest that the use of the subperiosteal tunnel technique can exhibit a reduced incidence of wound dehiscence.^5^ This approach is documented in clinical literature as a preventive measure to avoid contact between the incision lines and the graft material. Another approach is the vestibular incision,^23^ a strategy particularly recommended for titanium CAD/CAM titanium meshes.^24^, ^25^ In our study, a notably low dehiscence rate was observed, potentially attributable to the inherent healing properties of autogenous grafts, which possess osteogenic, osteoinductive, and osteoconductive characteristics. Nevertheless, flap passivation^26^ and surgical experience remain pivotal factors influencing uncompromised healing.

The literature on vertical iliac crest block grafts reveals varying rates of resorption, with some studies indicating higher rates,^27^ and others reporting long‐term stability.^14^ In our study, emphasis was placed on utilizing the cortical portion of the iliac crest as graft material due to its tendency for lower resorption compared to cancellous bone portions. Because bone resorption occurs with bone remodeling, initial healing results in the most pronounced bone loss. However, after implant placement and prosthetic loading, marginal bone levels remain stable. This was confirmed by the long‐term data of this study. However, the indication treated may play an important role. In edentulous patients, the remodeling process may be different because mucosa‐supported temporaries may increase the healing and resorption process. An additional factor contributing to long‐term stability is the soft tissue situation. The primary wound closure with its periosteal‐releasing incisions leads to a shift of the mucogingival junction. Given the necessity for keratinized peri‐implant soft‐tissue, supplementary procedures such as vestibuloplasty with free gingival grafts from the palate become imperative.^28^

This study has inherent limitations as it is an observational study rather than a randomized controlled clinical trial. Nevertheless, the decision to graft either outside or inside the bone contour is defect‐specific, making randomization impractical. It is crucial to acknowledge that bone regeneration is inherently specific to the nature of the defect. Consequently, some heterogeneity in the data exists, as the vertical defects, even when categorized using the Terheyden classification, are not entirely identical. Despite the challenges posed by variability in defect characteristics, the Terheyden classification effectively categorizes defects. However, it is noteworthy that many studies on graft procedures lack comprehensive descriptions of the type and size of treated bone defects. In contrast, this study employed multiple parameters for analysis and evaluated outcomes using a diverse set of criteria.

## 5 CONCLUSION

Within the limitations of this study, the use of vertical bone grafts, particularly with iliac crest block grafts, proved to be a reliable and an effective treatment option for bone augmentation. This approach successfully generates sufficient bone for the placement of dental implants. The observed vertical bone gain in the specified defect types was notably high, coupled with a low complication rate. Notably, block grafts placed outside the bone contour yielded a higher vertical bone gain without increasing marginal bone loss or complications. The study did not demonstrate a significant impact of larger bone grafts on marginal bone loss. This suggests that the method may be well‐suited for achieving reliable results in the regeneration of defects situated outside the bone contour.

## AUTHOR CONTRIBUTIONS

CM: Concept/Design, Data analysis/interpretation, Drafting article, Approval of article. CB: Data analysis/interpretation, Statistics, Critical revision of article, Approval of article. OR: Data interpretation, Critical revision of article, Approval of article. JH: Data interpretation, Critical revision of article, Approval of article. HLW: Data interpretation, Critical revision of article, Approval of article. KJH: Data collection, Data interpretation, Drafting article, Critical revision of article, Approval of article. All authors agree to be accountable for all aspects of the work, they will ensure that questions related to the accuracy or integrity of any parts of the work are appropriately investigated and resolved.

## CONFLICT OF INTEREST STATEMENT

The authors declare no conflict of interest.

## Data Availability

The data that support the findings of this study are available from the corresponding author upon reasonable request.

## APPENDIX A

**TABLE A1:** Linear mixed effect model for outcome “Graft height” (*n* = 197).

	Estimate	Lower 95% CI	Upper 95% CI	*p*‐value
(Intercept)	7.243	−0.97	14.401	0.07
Implant length	0.637	0.322	1.062	<0.001
Implant diameter	−0.529	−1.622	0.503	0.334
Age at augmentation	0.019	−0.026	0.063	0.449
Number of missing teeth	−0.199	−0.418	0.016	0.101
Indication trauma	0			
Indication atrophy	−1.953	−3.481	−0.348	0.025
Indication cleft palate	−1.708	−3.585	0.189	0.102
Indication pathology	−1.081	−3.085	0.881	0.321
BMI	0.036	−0.094	0.169	0.616
Maxilla	0			
Mandible	−1.245	−2.307	−0.091	0.037

**TABLE A2:** Linear mixed effect model for outcome “Graft width” (*n* = 197).

	Estimate	Lower 95% CI	Upper 95% CI	*p*‐value
(Intercept)	4.812	−0.052	9.238	0.047
Implant length	0.017	−0.177	0.24	0.872
Implant diameter	0.399	−0.222	1.067	0.226
Age at augmentation	0.016	−0.011	0.044	0.275
Number of missing teeth	−0.104	−0.233	0.022	0.138
Indication trauma	0			
Indication atrophy	0.368	−0.591	1.326	0.478
Indication cleft palate	−0.561	−1.724	0.59	0.369
Indication pathology	0.573	−0.677	1.8	0.394
BMI	−0.008	−0.086	0.072	0.857
Maxilla	0			
Mandible	−0.178	−0.872	0.537	0.64

**TABLE A3:** Linear mixed effect model for outcome Marginal bone loss mesial (*n* = 197).

	Estimate	Lower 95% CI	Upper 95% CI	*p*‐value
(Intercept)	0.6029	−1.7576	2.9635	0.6054
Implant length	−0.0628	−0.1620	0.0364	0.1988
Implant diameter	0.1232	−0.1239	0.3704	0.3102
Age at augmentation	0.0053	−0.0042	0.0148	0.2623
Number of missing teeth	−0.0004	−0.1210	0.1203	0.9947
Indication trauma	0			
Indication atrophy	−0.1093	−0.4752	0.2565	0.5448
Indication cleft palate	0.2237	−0.3418	0.7891	0.4094
Indication bone pathology	−0.2596	−0.6481	0.1289	0.1775
BMI	−0.0163	−0.0508	0.0182	0.3357
Maxilla	0			
Mandible	0.5019	0.1533	0.8505	0.0063
Graft length (graft level)	−0.0048	−0.0332	0.0236	0.7271
Graft width (graft level)	0.0595	−0.1890	0.3079	0.6053
Graft height (graft level)	−0.0301	−0.1254	0.0652	0.5203

**TABLE A4:** Linear mixed effect model for outcome Marginal bone loss distal (*n* = 197).

	Estimate	Lower 95% CI	Upper 95% CI	*p*‐value
(Intercept)	0.6891	−2.2039	3.5822	0.6292
Implant length	−0.0611	−0.1930	0.0709	0.3464
Implant diameter	0.1023	−0.2975	0.5022	0.6007
Age at augmentation	0.0063	−0.0037	0.0163	0.2049
Number of missing teeth	0.0056	−0.1174	0.1286	0.9243
Indication trauma	0			
Indication atrophy	0.0961	−0.3285	0.5207	0.6459
Indication cleft palate	0.3483	−0.1322	0.82 s88	0.1411
Indication bone pathology	−0.0058	−0.5148	0.5031	0.9811
BMI	−0.0273	−0.0635	0.0090	0.1321
Maxilla	0			
Mandible	0.4760	0.0982	0.8537	0.0153
Graft length (graft level)	−0.0056	−0.0346	0.0233	0.6899
Graft width (graft level)	0.0975	−0.1718	0.3667	0.4429
Graft height (graft level)	−0.0448	−0.1356	0.0460	0.3165
